# Microsurgical central lymphatic reconstruction—the role of thoracic duct lymphovenous anastomoses at different anatomical levels

**DOI:** 10.3389/fsurg.2024.1415010

**Published:** 2024-05-17

**Authors:** Andrea Weinzierl, Lisanne Grünherz, Gilbert Dominique Puippe, Ralph Gnannt, Donata von Reibnitz, Pietro Giovanoli, Diana Vetter, Ueli Möhrlen, Moritz Wildgruber, Andreas Müller, Claus Christian Pieper, Christian Alexander Gutschow, Nicole Lindenblatt

**Affiliations:** ^1^ Department of Plastic Surgery and Hand Surgery, University Hospital Zurich (USZ), Zurich, Switzerland; ^2^ Institute of Diagnostic and Interventional Radiology, University Hospital Zurich (USZ), Zurich, Switzerland; ^3^ Faculty of Medicine, University of Zurich (UZH), Zürich, Switzerland; ^4^ Department of Visceral and Transplant Surgery, University Hospital Zurich (USZ), Zurich, Switzerland; ^5^ Department of Pediatric Surgery, University Children’s Hospital Zurich, Zurich, Switzerland; ^6^ Department of Radiology, LMU University Hospital, LMU Munich, Munich, Germany; ^7^ Department of Neonatology and Pediatric Intensive Care, Children’s Hospital, University of Bonn, Bonn, Germany; ^8^ Department of Radiology, University of Bonn, Bonn, Germany

**Keywords:** central lymphatic reconstruction, lymphatic surgery, microsurgery, robotic microsurgery, lymphovenous bypass, thoracic duct lymphovenous bypass, thoracic duct anastomosis

## Abstract

**Introduction:**

In recent years advances have been made in the microsurgical treatment of congenital or acquired central lymphatic lesions. While acquired lesions can result from any surgery or trauma of the central lymphatic system, congenital lymphatic lesions can have a variety of manifestations, ranging from singular thoracic duct abnormalities to complex multifocal malformations. Both conditions may cause recurrent chylous effusions and downstream lymphatic congestion depending on the anatomical location of the thoracic duct lesion and are associated with an increased mortality due to the permanent loss of protein and fluid.

**Methods:**

We present a case series of eleven patients undergoing central lymphatic reconstruction, consisting of one patient with a cervical iatrogenic thoracic duct lesion and eleven patients with different congenital thoracic duct lesions or thrombotic occlusions.

**Results:**

Anastomosis of the thoracic duct and a nearby vein was performed on different anatomical levels depending on the underlying central lymphatic pathology. Cervical (*n* = 4), thoracic (*n* = 1) or abdominal access (*n* = 5) was used for central lymphatic reconstruction with promising results. In 9 patients a postoperative benefit with varying degrees of symptom regression was reported.

**Conclusion:**

The presented case series illustrates the current rapid advances in the field of central microsurgical reconstruction of lymphatic lesions alongside the relevant literature.

## 1 Introduction

**Figure 1:**
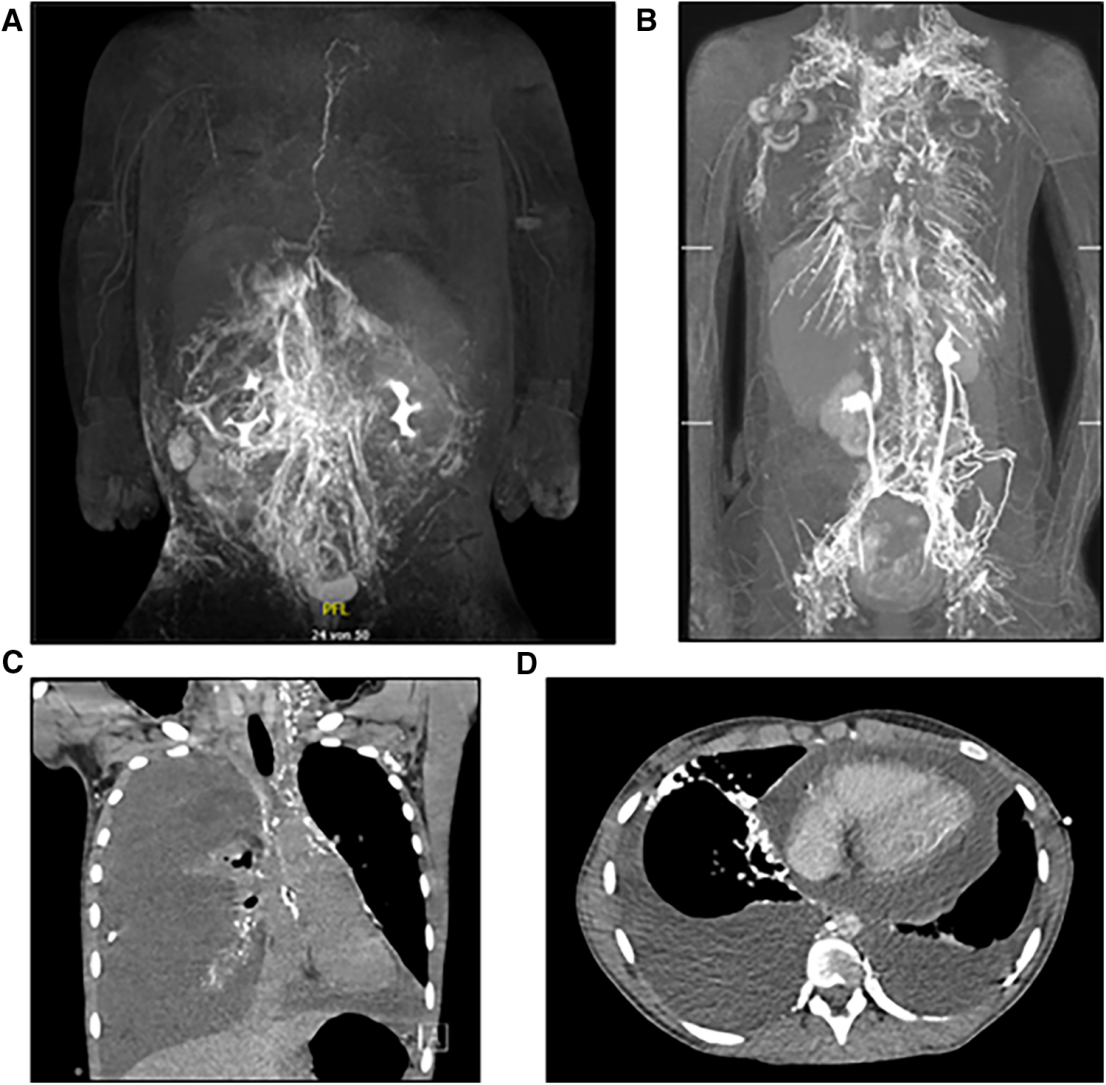
(**A**) MR-lymphangiography of a thrombotic occlusion at the left venous angle in a 6-month-old female patient as an example of an acquired central lymphatic flow disorder. (**B**) MR-lymphangiography of a 19-year-old patient with significant reflux in the context of a lymphatic drainage disorder, the congenital lesion is caused by a 13q deletion. (**C**) Exemplary CT-image of a recurrent chylopericardium. (**D**) Exemplary CT-image of a recurrent bilateral chylothorax.

Pathologies affecting the central lymphatic pathways include congenital conditions (e.g., thoracic duct (TD) malformations, central conducting lymphatic anomalies (CCLA) or Gorham-Stout syndrome) as well as acquired conditions (e.g., iatrogenic TD injuries) (Figures 1A, B) (1). These pathologies may lead to a plethora of symptoms including pleural effusion, pericardial effusion, ascites, organ-specific leaks, lymphedema, anasarca or a combination thereof with devastating effects on the patients' quality of life and life expectancy (2). Symptoms may result from lymphatic dysfunction, stenosis or discontinuity of the lymphatic system and lead to retrograde lymphatic leakage into body cavities (Figures 1C, D).

The first line treatment of TD lesions is usually a conservative approach with a low fat diet or total parenteral nutrition (3). Minimally invasive treatment modalities, such as percutaneous TD embolization as well as therapeutic lymphangiography are additional treatment optiona and have been reported with good success rates (4–6). However, complications such as distant lymphatic congestion have been shown to occur in patients following a disruption of the lymphatic drainage by means of TD embolization (7–9). Especially in patients with liver cirrhosis and cardiac insufficiency TD function has to be considered. In this particular subset of patients, restoration of lymphatic circulation may be necessary to reduce interstitial edema, improve hepatic microcirculation as well as cardiac output. Stenting of the TD has been proposed as an alternative non-ablative approach, but some of the different techniques remain difficult to perform with failure rates of up to 33% (10–12). The surgical management of TD lesions by thoracic duct-vein (TD-vein) anastomosis represents another approach (13), that dates back to 1953 (14). Reconstructive microsurgery of the central lymphatic system was long considered almost unfeasible due to the lack of suitable visualization and instruments that enable a microsurgical procedure in a deep cavity. However, thanks to recent technical advancements, TD-vein anastomoses have gained new significance for patients with aquired and congenital lymphatic lesions.

**Figure 2:**
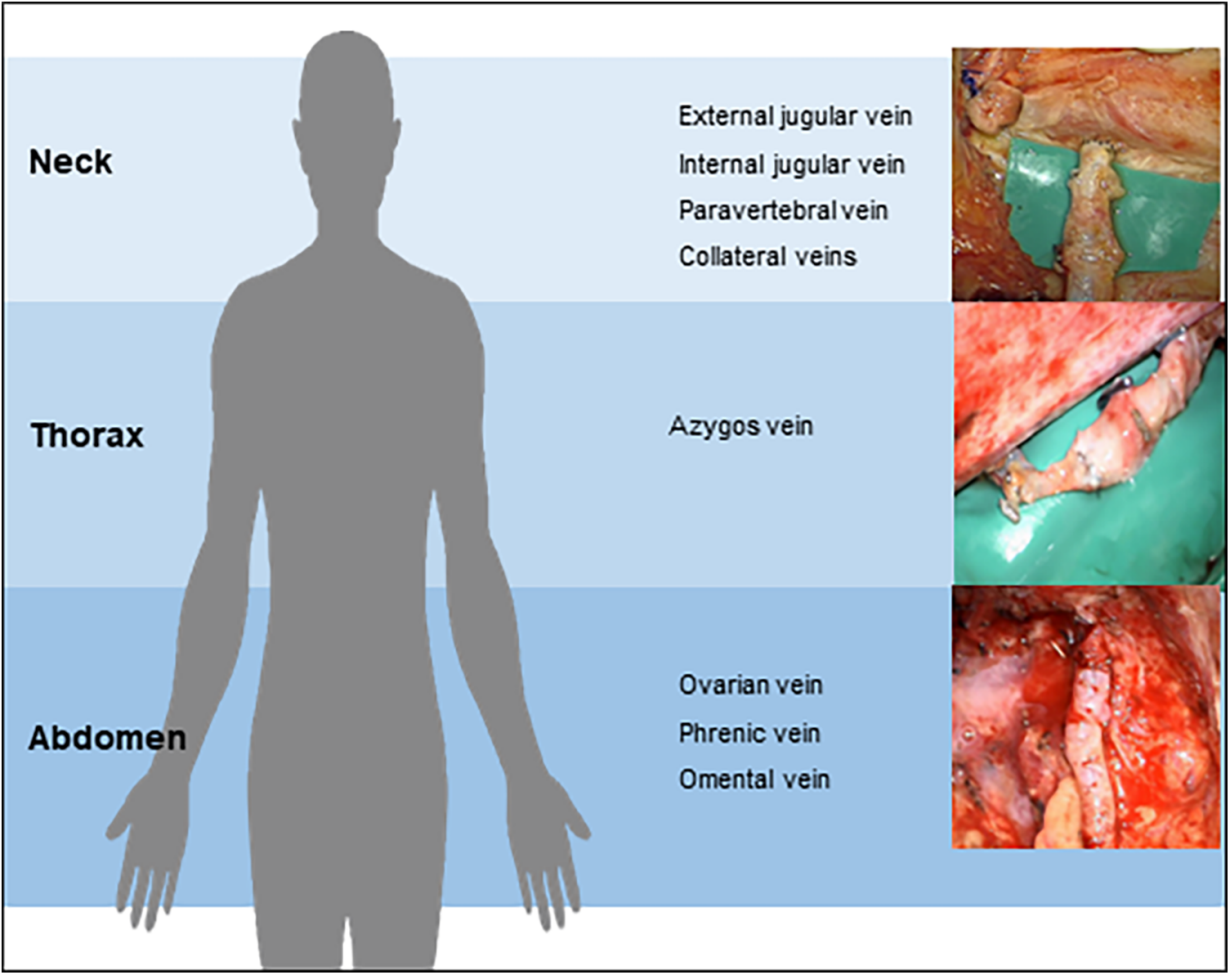
Central lymphatic reconstruction may be performed on different anatomical levels depending on the underlying lesion.

In the following we report on eleven cases with central lymphatic lesions of different aetiology and anatomical levels who received microsurgical lymphatic reconstruction after conservative treatment and embolization had failed (Figure 2). All surgeries were performed by the senior author at our institution. Depending on the anatomical site of the TD-vein anastomosis, the operation was performed in collaboration with a visceral surgeon.

## 2 Patients and methods

We conducted a retrospective study including patients with acquired or congenital lesions of the central lymphatic system receiving microsurgical central lymphatic reconstruction at the Department of Plastic Surgery and Hand Surgery of the University Hospital Zurich between October 2018 to January 2024. Approval was given by the Cantonal Ethics Committee of Zurich, Switzerland (Ethical approval Nr.: 2023-01091). Written consent was obtained from all patients or their legal guardian in case of minors. Additional consent was obtained from patients whose pre-, intra- and postoperative images are shown.

## 3 Results

**Figure 3:**
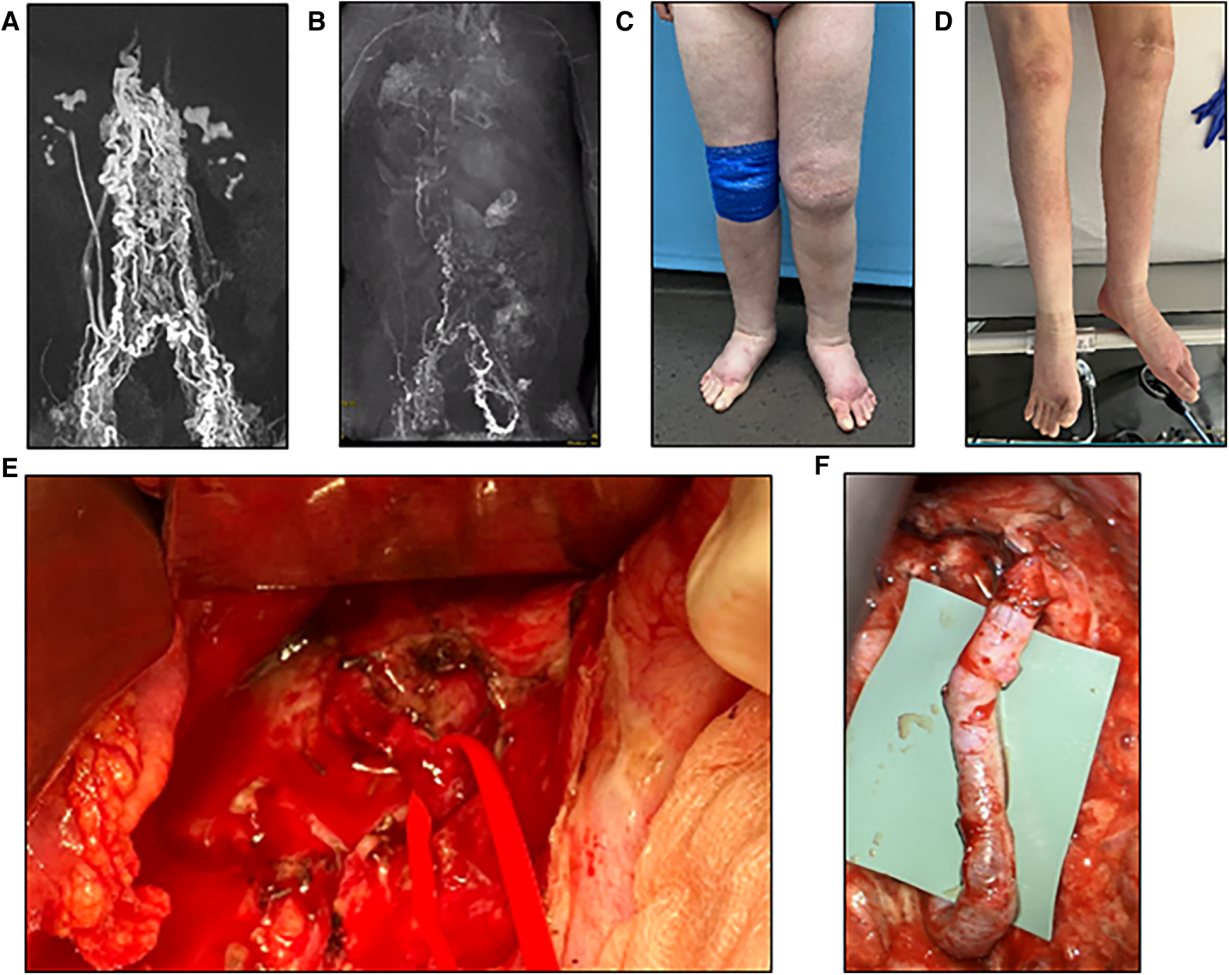
(**A**) Preoperative MR-lymphangiography of a 19-year-old female patient showing a massive dilatation of the lymphatic system. (**B**) Two months postoperative MR-lymphangiography showing a rapid flow of contrast agent into the venous system and a significantly reduced lymphatic dilatation. (**C**) Preoperative photographic images of the severe lymphedema of the lower legs in the same patient. (**D**) Two weeks postoperative the patient has lost 10 kg and does not wear compression garments continuously. (**E**) Intraoperative visualization of the thoracic duct after mobilization of the duodenum/pancreas via laparotomy. (**F**) Lymphovenous anastomosis of the thoracic duct to the right ovarian vein displaying a chylous washout in the vein.

**Figure 4:**
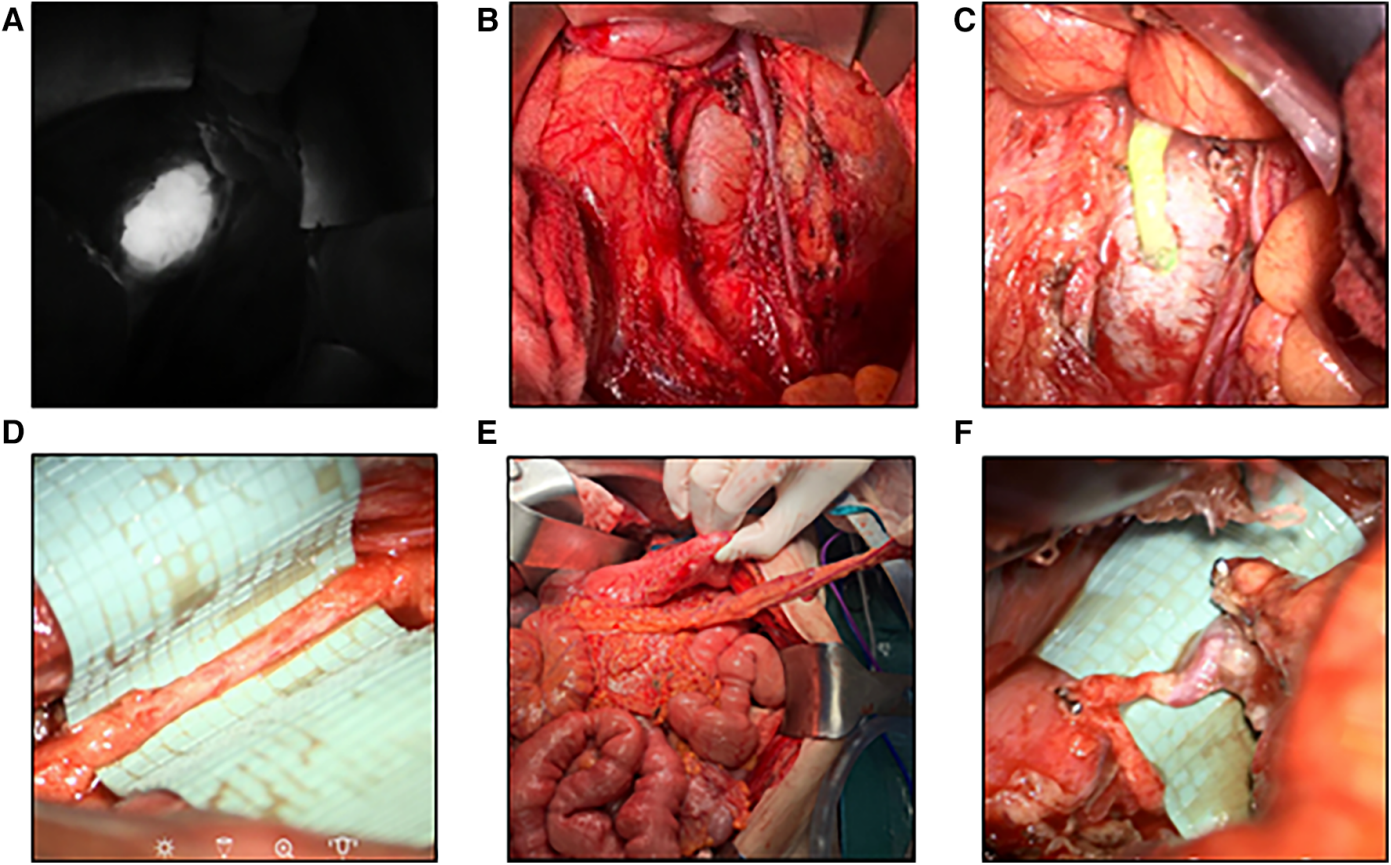
Robotic-assisted anastomosis at the abdominal level (**A**) visualization of an aneurysmatic dilation of the central lymphatic pathways after ICG-injection. (**B**) Surgical visualization of the dilation by means of laparotomy (15). (**C**) Patent lymphovenous anstomosis of the left ovarian vein to the dilation as visualized by an influx of ICG from the cyst into the vein (15). (**D**) Image of a hypoplastic thoracic duct (1.5 mm) below the diaphragm. (**E**) Surgical preparation of a pedicled omental flap to reach the hypoplastic thoracic duct for anastomosis. (**F**) Lymphovenous anastomosis of the thoracic duct to the gastroepiploic vein of the omental flap.

Eleven patients with acquired or congenital lesions of the central lymphatic system received microsurgical central lymphatic reconstruction (Table 1). The age of the patients varied from six months to 60 years. While one patient presented with a cervical iatrogenic TD lesion following neck dissection, the remaining patients were diagnosed with different congenital TD lesions or thrombotic occlusions. Of those, six patients suffered from CCLA with chylothorax, chylous ascites and/or chylopericardium, depending on the location of impaired central lymphatic drainage. One patient developed severe protein-loosing enteropathy (PLE), plastic bronchitis and lower extremity lymphedema with an abruption of the TD below the diaphragm following Fontan surgery with multiple cardiac procedures in the past (Figure 3). Another patient was diagnosed with a retroperitoneal central lymphatic cyst causing abdominal pain and reduced physical capacity with fainting after walking short distances (Figures 4A–C) (15). Two infants were diagnosed with severe bilateral chylothorax, one of them additionally suffered from chylous ascites, anasarca, PLE and multiple venous thromboses. While magnetic resonance lymphangiography (MRL) showed a lack of lymphatic drainage of the head as well as massive intestinal reflux, the exact etiology remained unknown.

**Table 1:** Patients treated for central lymphatic lesions.

Patient (age in years)	Preoperative condition	MRL	Duration of conservative treatment (months)	Previous intervention/surgery	Surgical access	Surgery	Symani surgical system	Follow-up	Outcome
1 (52) M	Iatrogenic lesion of TD with chylous fistula	None	1.5 months	None due to absent cisterna chyli	Neck	End-to-end anastomosis of TD and EJ	No	5 years	No recurrence of lymph leak
2 (52) F	CCLA with bilateral chylothorax	TD stenosis in the mediastinum with upstream dilatation of the TD	6 months	LA: recanalization impossible	Median laparotomy	Intraabdominal end-to-end anastomosis of TD and LIPV	No	4 years	Complete remission
3 (6 months) F	Bilateral chylothorax, chylaskos anasarca including head and neck and PLE, multiple venous thromboses	Lack of lymphatic drainage of the head, massive intestinal reflux	6 months	Multiple attempts of catheter-based lysis; multiple interventional thrombectomy and stenting	Neck	Antero- and retrograde end-to-end anastomosis of TD and collateral vein, 1× cervical LVA	No	6 weeks	PLE remission, improvement of anasarca and chylothorax; death during thrombolysis 6 weeks postop
4 (19) F	CCLA with recurrent bilateral chylothorax	Dysplastic TD and aberrant lymphatic drainage along the abdominal wall and chest	5 years	None	Chest wall	1× LVA and MLL lateral chest wall	No	1 year	Persistence of chlyothorax
5 (21) F	CCLA with anasarca and bilateral chylothorax	Occlusion of TD at the subclavian angle	10 years	None	Neck	3× LVA at the neck	No	10 months	Regression of symptoms with improvement of anasarca and chylothorax
6 (47) F	Lower extremity edema, abdominal pain and reduced physical capacity	Aneurysmal dilatation of the left paramedian lymphatic duct and dilated lymphatic cisterns along the iliac lymphatic pathways	NA	LA: embolization	Median laparotomy	Intraabdominal end-to-side anastomosis of left OV with lymphatic cyst	Yes	7 months	Regression of symptoms; MRL proved drainage from the aneurysmal dilatation into the ovarian vein
7 (19) F	PLE, plastic bronchitis and lower extremity edema after Fontan surgery	Abruption of TD below the diaphragm, hepato-eneral lymphatic reflux	NA	LA: recanalization	Median laparotomy	Intraabdominal end-to-end anastomosis of TD to right OV	No	7 months	Regression of lower extremity edema
8 (10) M	CCLA with bilateral chylothorax	TD abruption, thoracic lymphatic malformation on the right and lymphatic reflux on the left	2 months	LA: embolization	Median laparotomy	LVA between subdiaphragmal lymph vessel and omental vein; MLL	Yes	5 months	Persistence of chylothorax; Additional interventional embolization
9 (60) F	CCLA with bilateral chylothorax and lower extremity lymphedema	TD not detectable, abruption of retroperitoneal lymphatic pathways at the level of lumbar vertebra 2	30 years (for lower extremity lymphedema)	None	Median laparotomy	Intraabdominal end-to end anastomosis of TD and right GEV, para-aortal and iliac LVA on the left	Yes	3 months	Regression of chylothorax
10 (17) F	CCLA with bilateral chylothorax and chylopericardium	atresia of the TD at the cervical angle	4 months	LA: embolization	Right-sided thoraco-tomy	End-to-end anastomosis of TD and AV; MLL	Yes	2 months	Partial regression of symptoms with increased physical fitness
11 (8 months) M	Bilateral chylothorax	TD stenosis at the level of the angulus venosus sinister, multiple venous thrombosis	8 months	LA: recanalization	Neck	End-to-end anastomosis of TD and branch of the left EJ	Yes	1 months	Reduction of chylothorax, intermittend clamping of drains

CCLA, central conducting lymphatic anomaly; TD, thoracic duct; AV, azygous vein; EJ, external jugular vein; OV, ovarian vein; GEV, gastroepiploic vein; LA, conventional lymphangiography; PLE, protein losing enteropathy; LM, lymphatic malformation; LIPV, left inferior phrenic vein; MRL, MR Lymphangiography.

Except in the patient with iatrogenic TD lesion, MRL was performed in all remaining patients to localize the underlying pathology of the central lymphatic system. Regardless of the etiology, all patients with lymphatic leakage initially received conservative treatment for at least six weeks (range 6 weeks–10 years). If conservative treatment failed, in almost all patients with CCLA either recanalization or embolization by conventional lymphangiography (LA) was performed. In some cases, interventional embolization of the refluxing lymphatic vessels or even TD embolization was not performed because it would have entailed the risk of worsening the chylous effusion.

**Figure 5:**
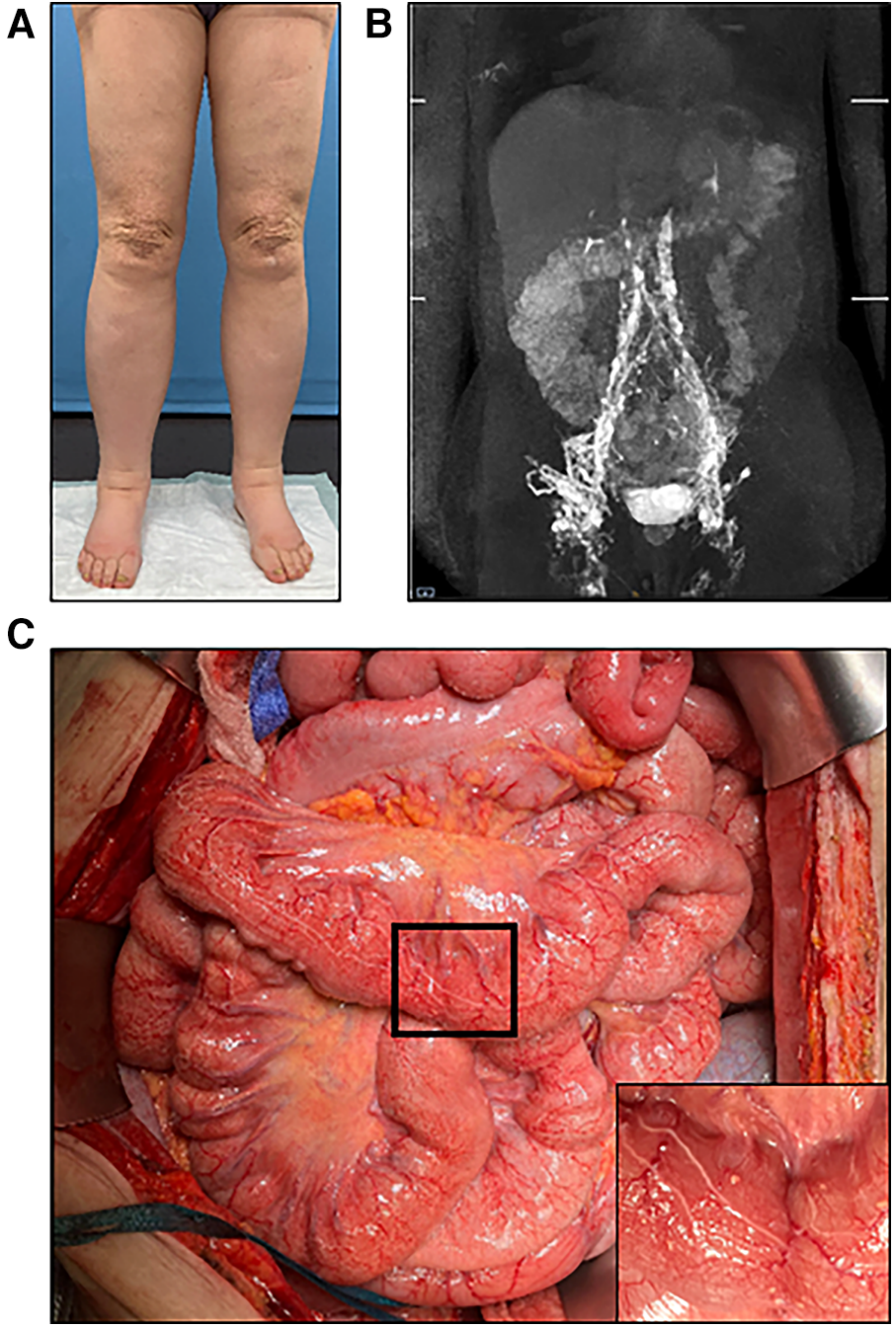
(**A**) Preoperative photographic images of severe lymphedema of the lower extremities in a 60-year-old female patient. (**B**) MR-lymphangiography reveals central lymphatic flow disorder. (**C**) During laparotomy, dilation of the intestinal lymphatic vessels is clearly visible. Higher magnification image of the area marked by black frame in the inset.

Any surgical treatment was carefully planned on the basis of the MRL in an interdisciplinary approach involving multiple case discussions. The location of the TD-vein anastomosis was based on the suspected localization of the impairment of lymphatic drainage, the suspected lymphatic anatomy and the availability of adjacent veins for reconstruction. Access to the central lymphatic system was achieved at different anatomical levels (Figures 4, 5). With regard to the underlying central lymphatic lesion, a cervical (*n* = 4), thoracic (*n* = 1) or abdominal access (*n* = 5) was used to reach the TD. Intranodal inguinal indocyanine green (ICG) injection by interventional radiology at the beginning of the surgery in combination with a near infrared camera was used to localize the central lymphatic pathways. After localization of the lesion, an anastomosis of the TD and a nearby vein was performed. The Symani Surgical System**®** was used in five patients to anastomose the vessels [Medical Microinstruments (MMI), Inc., Wilmington, USA].

## 4 Case presentation

We present two exemplary cases in more detail to elucidate the typically extended number of treatments patients with central lymphatic flow disorders receive.

### 4.1 Case 1

**Figure 6:**
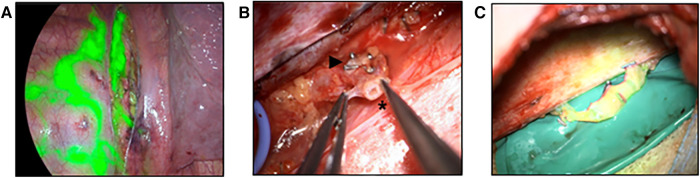
Anastomosis of the thoracic duct to azygos vein (**A**) thoracoscopy after ICG injection into groin lymph nodes. The image shows the thoracic duct (central) as well as multiple pleural collaterals (left). (**B**) Surgical preparation of the thoracic duct (*) and the azygos vein (►). (**C**) Patent lymphovenous anastomosis as verified by the flow of ICG into the azygos vein.

We report on a 60-year-old patient with lymphedema of her lower extremities present for the past 30 years, initially treated conservatively (Figures 5). The patient required laparoscopic hernia surgery three years prior to her presentation at our institution as a consequence of surgical hysterectomy. After hernia surgery, she developed delayed wound healing with persistent lymphatic secretion from the umbilical wound. Afterwards, her lymphedema progressed and spread from the lower extremities to the abdomen and hips. In addition, the patient was diagnosed with a chylothorax, which had to be treated by video-assisted thoracoscopic surgery. MRL revealed a disruption of the central lymphatic flow. Thus, a medium chain triglyceride (MCT) diet was established. However, this only led to a partial regression of the symptoms. Subsequent digital subtraction angiography of the lymphatic pathways showed an abruption of the lymphatic flow at the level of the second lumbar vertebrae (Figure 6). Due to repeated chylothorax, thoracic drainages were placed bilaterally in addition to the continued conservative treatment of the lower extremity lymphedema. The thoracic drainages produced ∼400–500 ml/24 h on average despite adjuvant treatment with propranolol. The decision was made to perform central lymphatic reconstruction in collaboration with the visceral surgery team.

Access was established by median laparotomy to visualize the lumbar left para-aortic and left iliac lymphatic pathways by mobilization of the colon. In addition, mobilization of the liver and access to the retroesophageal space was established to visualize the TD in the lower mediastinum. Injection of ICG at the mesenteric root and into the lymphatic tissue at the coeliac trunk was performed in order to identify the TD in the lower mediastinum. This allowed the senior author to perform a lymphovenous anastomosis between the hypoplastic TD (diameter 1.5 mm) and the slightly larger right gastroepiploic vein. Additionally, robot-assisted lymphovenous anastomoses between the lumbar left para-aortic and left iliac lymphatic vessels and the left ovarian vein were performed using the Campisi technique. To execute robot-assisted anastomosis, patient positioning was adapted intraoperatively to accommodate the angled robotic arms. Of note, the use of the microsurgical robot requires practice and was utilized due to the familiarity of the senior surgeon with this system in this challenging case.

Three months postoperatively the patient showed a clinical improvement with a markedly diminished volume of fluid in her thoracic drainages and intermittent clamping of the drains without significant fluid accumulation.

### 4.2 Case 2

We present the case of a 17-year-old patient with CCLA and bilateral chylothorax as well as chylopericardium diagnosed 6 months earlier. In the context of increasing dyspnea, one year of asthma treatment and a scooter accident, the effusions were detected by a CT scan. Subsequent MRL and conventional lymphangiography revealed a central disruption of lymphatic drainage. Imaging revealed a high suspicion of a lack of lymphatic drainage at the venous angle with pronounced collaterals and formation of pericardial and pleural effusions. While the TD could still be identified in the lower third of the mediastinum, no cisterna chyli could be visualized and imaging showed only several fine lymphatic vessels.

The patient and her parents chose to attempt a reconstruction of the lymphatic drainage before considering alternative treatments such as pericardial adhesion or pleurodesis. Therefore, reconstruction of the central lymphatic flow by means of lymphovenous anastomosis was performed (Figure 6). After thoracoscopic dissection of the TD and the azygos vein as well as ICG injection to confirm correct TD identification, surgical access was established through right posterolateral thoracotomy in the 6th intercostal space by the visceral surgeon. In order to perform TD-vein anastomosis to the azygos vein, TD transection was performed in the lower third of the thorax with subsequent ligation of the proximal stump. Microsurgical anastomosis was completed and patency of the anastomosis was confirmed due to immediate drainage of ICG into the azygos vein. Additional ligation of lymph collectors running towards the pleura was performed without complication.

Two months postoperatively, the patient's symptoms, in particular her dyspnea, had improved with a significant regression of the pericardial effusion and the right sided chylothorax as well as a complete regression of the left sided chylothorax, allowing a normal school attendance and climbing of two floors of stairs without dyspnea. Additional interventional treatment to further reduce the pleural effusion with suspected established lymphatic collaterals in the lower thorax is planned.

## 5 Discussion

Since the access to the anatomically deep central lymphatic system remains surgically challenging, central lymphatic reconstruction has gained clinical importance only in recent years due to technical advancements. It is currently regarded as individual treatment approach after conservative therapy and interventional treatments have failed. Often pesistent chylous effusions into variuos body caveties have been present for months to years without prospect for healing.

Conservative therapy includes dietary or pharmacological treatment. Besides MCT diet, pharmacologic interventions such as the use of octreotide, propanolol, sirolimus, sildenafil or trametinib have shown effectiveness (16–20). If adequate symptom control cannot be achieved through conservative treatment, lymphangiography-guided interventions are considered the treatment of choice for central lymphatic lesions. These interventions allow for different treatment modalities depending on the entity of the lesion with success rates of up to 80%–90% and a complication rate of 7% (21–23). In this context, the use of ICG-lymphography and lymphangiography has proven invaluable for diagnostic as well as therapeutic use (24–26). As added oil contrast agents used to perform lymphangiography may cause lymphatic injuries, embolization of the leakage point of lymphatic fluid in chylothorax or chyloabdomen may occur in addition to the visualization of the lymphatic pathways. However, therapeutic occlusion of the TD may carry the risk for the development of lower extremity lymphedema, protein-losing enteropathy or increased lymphatic reflux with a fistula at another site (27). These complications should also be considered if microsurgical ligation of the central lymphatic pathways is taken into consideration as a treatment option. Thus, reconstructive microsurgical treatment needs to be considered as well, to optimize each patients treatment. Due to the rarity of central lymphatic lesions, we advocate a low-threshold referral to specialized lymphatic centers for the diagnosis and treatment in such cases.

Traumatic or iatrogenic transection of the TD usually leads to persistent leakage of chyle into wounds or body cavities. If a cutaneous chyle fistula drains into an open wound, reconstructive surgery can and should be considered as part of the wound revision. This approach needs to be be taken into consideration, especially if interventional treatment is not feasible, for instance due to absence of a cisterna chyli. TD-vein anastomosis in the retroperitoneal area or at the neck, depending on the location of the chyle leak, have been used successfully (28–31). As central lymphatic lesions encompass a large spectrum of pathologies and lesions, different surgical techniques need to be considered for an optimal treatment. Arguably the best postoperative results seem to be achieved by performing TD anastomosis to a nearby vein, as illustrated by a growing number of case reports (28, 29, 32–35).

We herein propose a patient-specific treatment plan choosing different recipient vessels depending on the location and anatomy of the lesion. This flexibility facilitates individual surgical solutions that can be adapted to the specific type of central lymphatic lesions, optimizing the overall outcome.

However, the treatment of congenital central lymphatic disorders, in particular those involving malformed lymphatic vessels, remains very challenging. In many cases there is a history of months to years of high volume chyle leaks via body cavities, e.g., pleural, peicardial or peritoneal effusions. The establishment of a drainage into the venous system will provide a new pathway for the chylous fluid to enter the bloodstream. However, due to the higher pressure within the venous system compared to the the pressure in the body cavity, where drains are often still present, it may take several weeks for the system to find a new equilibrium. In addition, an extended network of collateral lymphatic vessels may be present reducing the flow over the newly created lymphovenous anastomosis. Adjunct interventional treatment has been shown to be beneficial to obliterate the aberrant lymphatic pathways.

Besides end-to-end TD anastomosis or TD-vein anastomosis as described in the present case series, other surgical techniques such as lymph node-to-vein anastomosis to treat chylothorax have been suggested (36). Moreover, performing multiple LVAs on the lower extremity has been suggested to bypass proximal lymphatic impairment in patients suffering from recurrent chylous and lymphatic ascites (15, 37). Another microsurgical treatment approach for refractory ascites is the creation of LVAs between lymphatic vessels and veins of the greater omentum in the abdomen (38). Though larger studies have not been performed, promising case reports with a good surgical outcome exist (13). Moreover, the use of new robotic microsurgical systems may lead to a more widespread and successful surgical management of these pathologies (Supplementary Video S1). For instance, in 2023 we reported on the successful use of the Symani® surgical system (Medical Microinstruments, Inc., Wilmington, USA) to treat a patient with central lymphatic dilation causing abdominal pain and severely reduced physical capacity (15). Besides enhancing the precision and endurance of the surgeon, the use of the microsurgical robot may enable surgical interventions in deeper anatomical planes that were so far difficult to access (39). Though additional costs for the robot itself and the associated consumables have to be considered, the use of the robotic system offers several benefits such as smaller surgical access sites or avoiding the use of longer and possibly less precise instruments. Due to the utilization of special manipulators, there is little difference between robotic and manual anastomosis regarding the movements of the surgeon, facilitating an intuitive use. Tilting of the patient may be of use to position the angled robotic arms during surgery. Overall, this technique may also be associated with other benefits such a faster reconvalescence due to the smaller incision sites in the future. Since the robotic system is free-standing it has been proven to be of particular benefit in the anastomosis of vessels less than 1 mm in small children, e.g., the thoracic duct in the neck, since movement of the operative field due to hand placement is avoided. Current limitations include a fixed angle of the arms of the robotic system occasionally requiring the use of a larger incision, which may be overcome in the future by design adjustments.

## 6 Conclusion

The present case series illustrates the possibility of a microsurgical reconstruction to treat central lymphatic lesions, in particular if congestion of the central lymphatic flow is present. Given the burden and the high morbidity of this complex pathology, microsurgical reconstruction offers a chance of symptom relieve with an acceptable perioperative risk for the patients. Due to the complex physiology of congenital central lymphatic anomalies with an impaired peristalsis and multiple sites of chylous leakage, treatment success can vary. We believe that the interdisciplinary co-operation with other specialties including visceral and thoracic surgeons as well as interventional radiologists is vital for successful microsurgical reconstruction, as described in the presented cases. Due to the rarity of these lesions, it is not possible to conduct large scale clinical studies, but future research may be able to provide further insight into this rapidly developing field, for instance by evaluating long term outcomes. Robotic-assisted microsurgery may significantly advance reconstruction of the central lymphatic system in the future.

## Data Availability

The datasets presented in this article are not readily available because Patient data protection. Requests to access the datasets should be directed to nicole.lindenblatt@usz.ch.

## Supplementary material

Supplementary Video S1 Video of a robotic assisted lymphovenous anastomosis of the lumbar lymphatic trunks to the ovarian vein.
